# Organizational challenges persist, and new research directions emerge in the study of burnout in healthcare: Bibliometric analysis

**DOI:** 10.1177/22799036251395259

**Published:** 2025-11-20

**Authors:** Carla W. Irigoyen-Amparan, Karen D. Gonzalez, Arunkumar Pennathur, Bibiana Mancera, Priyadarshini R. Pennathur

**Affiliations:** 1 Physical, Information and Cognitive Human Factors Engineering Research Laboratory, Industrial, Manufacturing and Systems Engineering Department, The University of Texas at El Paso, TX, USA; 2 Border Biomedical Research Center, The University of Texas at El Paso, TX, USA

**Keywords:** burnout, healthcare, organizational factors, workplace stressors, workplace aggression, COVID-19, emerging technology

## Abstract

**Background:**

Between 35% and 45% of nurses and 40%–54% of physicians in the United States experienced burnout over the past decade, underscoring the need to examine trends and patterns in healthcare burnout research to identify contributors and formulate recommendations. Our objectives were to (1) understand whether the problem of burnout is widespread and studied globally, (2) assess the extent of research collaboration, (3) examine the focus of healthcare burnout themes prior to 2019 and after 2019 and assess similarities between themes to identify persistent problems, and (4) assess differences in themes to identify new research directions triggered by COVID-19.

**Design and methods:**

We performed a literature search in Web of Science, followed by bibliometric and manual comparative analyses of publications data. We analyzed trends in publications, countries, and organizations where healthcare burnout was studied, constructed co-authorship networks, and evaluated theme similarities and differences between the periods.

**Results:**

Studies have investigated longstanding system and organizational problems, including poor workplace conditions and unsupportive leadership and management, as contributors to burnout. Research collaborations on healthcare burnout across countries have increased post-pandemic. Studies conducted after 2019 have investigated new research directions, including workplace adaptations, workplace aggression, and emerging technologies such as virtual reality.

**Conclusions:**

Our findings indicate that workplace conditions and organizational factors such as leadership and management remain persistent challenges, with workplace violence and workplace aggression increasingly associated with burnout. Design improvements to the work system and emerging technologies hold promise as interventions for preventing and mitigating burnout.

**Significance for Public Health:** Despite high prevalence of burnout among healthcare workers, understanding of persistent and system level factors that could lead to burnout remains limited. Many studies examine job-related or occupational factors in isolation, resulting in fragmented insights and siloed knowledge, while system-level contributors to burnout remain insufficiently integrated across research. In our study, we conducted a bibliometric analysis to document patterns and trends in healthcare burnout research, allowing us to synthesize existing work and highlight the factors most examined in the literature for their persistent association with healthcare burnout. Our findings show poor workplace conditions including increasing workplace violence and unsupportive leadership and management as persistent contributors to healthcare worker burnout, demonstrating the urgent need to examine organizational redesign as a mediator for healthcare worker burnout to address the serious public health implications burnout can bring to healthcare workers, patients, and healthcare organizations.

## Introduction

The 2019 report from the National Academies of Engineering and Medicine (NAEM) highlights that between 35% to 45% of nurses and 40% to 54% of physicians in the United States experience symptoms of burnout.^1^ Studies indicate that workers in the healthcare industry are more vulnerable to burnout than those in other occupations.^2^

Burnout, which is characterized by three dimensions—emotional exhaustion, depersonalization or cynicism, and a low sense of professional accomplishment^3^ as conceptualized by Maslach Burnout Inventory (MBI),^4^ has implications not only for healthcare workers (HCWs), but also for patients and healthcare organizations. Studies have shown that workers who experience workplace burnout are likely to develop clinical conditions such as diabetes^5^ and depressive disorders,^6^ and experience diminished well-being,^7^ highlighting potential risk factors for HCWs. Additionally, burnout is associated with a lower perceived quality of care.^7^ It can also lead to missed and unsafe care for patients.^8–10^ Research has demonstrated that clinicians experiencing burnout, with particularly high emotional exhaustion and depersonalization dimensions, are at least twice as likely to commit a significant medical error compared to those who are not burnt out, which could undermine the quality of the service provided.^1^ Additionally, factors such as workload, experience, and practice setting can also impact the relationship between burnout and patient safety. Burnout also presents organizational challenges in workforce preservation, quality of care, and healthcare system sustainability. Studies show that increased employee absenteeism and reduced productivity are associated with increased burnout, reducing organizational performance and increasing costs.^11,12^ The healthcare industry faces substantial turnover costs, with 2400 US physicians leaving annually.^13^ The turnover rates among nurses in other countries are also high, with 44.3% of nurses in New Zealand, 26.8% in the US, 19.9% in Canada, and 15.1% in Australia intending to leave the workforce.^14^ The World Health Organization estimates a global deficit of 18 million healthcare workers by 2030, which can lead to a public health crisis.^15^ These trends indicate the significant staffing shortages, partly due to stress and burnout.^16–21^

In examining contributors to burnout, earlier studies in the 90s focused primarily on emotional exhaustion^22^ and explored individual-level factors and coping strategies rather than organizational and system-level contributors.^23^ In the 2000s, there was an explicit focus on occupational settings and work environments. In particular, studies have examined the associations between burnout and workplace factors such as decreased control, lack of teamwork, inadequate resources,^24^ and workplace support, underscoring the crucial role of organizational factors.^22^ More recent studies during COVID-19 have expanded the focus to both individual and organizational factors, including longer work hours, sleep deprivation, job demands and resources, caring for severely ill patients, and increased administrative tasks,^25,26^ highlighting the associations between workplace adaptations needed for COVID-19, resulting working conditions, and corresponding work stressors and burnout.

Work stressors, such as work overload and working conditions, can affect burnout and worker well-being^27^ and may have intrinsic, demonstrable linkages to staffing shortages and challenges in workforce retention in healthcare, warranting additional investigations.^19,23,28–30^ For example, undesirable workplace conditions, such as workplace violence, have been rising recently,^31^ pointing to management and cultural problems in healthcare organizations requiring immediate attention. These undesirable work conditions need a multilevel approach that could be addressed through focus and prioritization on broader system level factors such as workload management, adequate staffing, organizational policy changes, leadership training and workplace safety improvements, and resources such as mental health support programs alongside individual interventions. Such interventions can target healthcare workers at risk of or experiencing burnout, leadership teams who need tools to assist their teams, and organizational decision makers who can help implement these system level changes.

Research on burnout began in the 1970s and the 1980s when the concept was first defined.^31^ By the 1990s, standardized measures such as the Maslach Burnout Inventory (MBI)^4^ – assessing emotional exhaustion, depersonalization, and personal accomplishment—enabled evaluative studies that identified individual and organizational contributors to burnout among healthcare workers. Other instruments, such as the Copenhagen Burnout Inventory, which assesses personal burnout, work-related burnout, and client-related burnout for use in different domains, were developed subsequently in the 2000s.^32^ In 2019, burnout was formally recognized as an occupational phenomenon by the World Health Organization International Categorization of Diseases.^33,34^ The US NAEM also issued a call in 2019 to address its growing impact. The COVID-19 pandemic further accelerated research into how work conditions and rapid workplace adaptations shaped burnout^23,28–30^ across clinical specialties. Despite this history, current bibliometric reviews remain limited, often focusing on specific roles, such as nursing.^34,35^ What is still missing is a comprehensive view of the global research landscape: which factors have persisted across decades, how research directions shifted pre- and post-COVID, and what systemic gaps remain. Addressing this gap is critical—without it, healthcare organizations risk directing resources toward surface-level symptoms rather than targeting the enduring, underlying causes of burnout.

Given this gap in knowledge, our aim was to conduct a bibliometric analysis to (1) understand whether the problem of burnout is widespread and studied globally by identifying publication, country, and organization trends where healthcare burnout is studied; (2) assess the extent of research collaboration by examining co-authorship networks; (3) examine the focus of healthcare burnout themes prior to 2019 and after 2019, and assess similarities between themes to identify persistent problems; and (4) assess differences in themes after 2019 to identify new research themes set in motion by COVID-19.

## Methods

### Overview of bibliometric analysis and preparation for analysis

Bibliometric analysis synthesizes literature to understand the “intellectual structure and emerging trends”^35^ and to understand research themes and identify collaboration patterns^36–38^ in a discipline. We used it to understand how research themes on burnout in healthcare have evolved. Bibliometric analysis is valuable for analyzing large volume of bibliographic data using tools such as VOSviewer.^39^ The reporting of our study conforms to the BIBLIO guidelines.^40^

#### Search strategy

**Figure 1.**
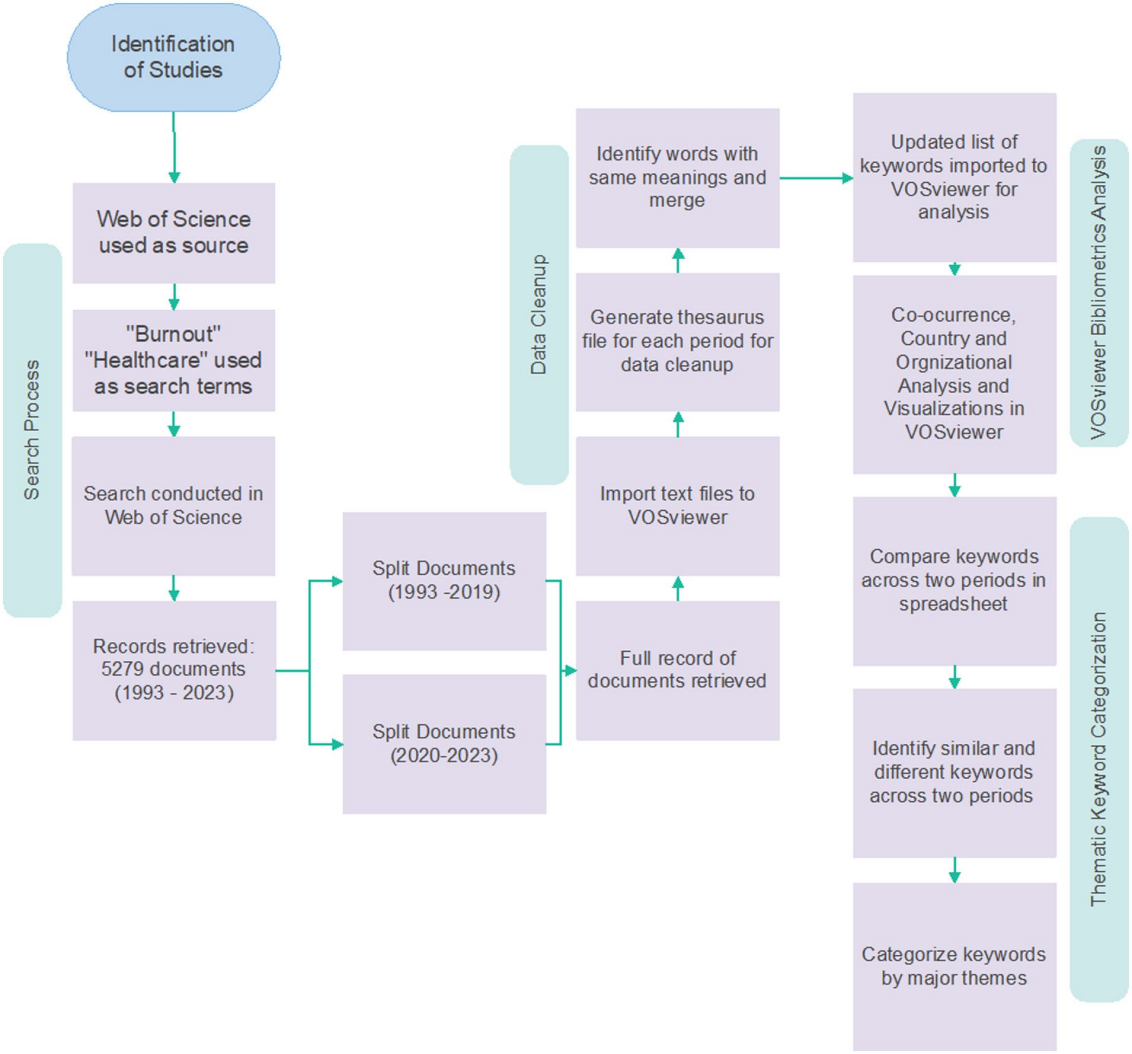
Process steps for bibliometrics analysis and keyword theme categorization. Source: Author’s own work.

We used Web of Science (WoS) for searching and retrieving literature. The steps of the search and analysis are shown in Figure 1. We chose Web of Science because it offers comprehensive citation data essential for bibliometric network analysis with consistent indexing and metadata quality. Web of Science is also compatible with VOSviewer for reliable data processing and visualization.

Authors 1 and 2 used “burnout” and “healthcare” with Boolean operator “AND” to retrieve publications. We only included publications written in English. We did not limit the type of organizations to any specific institution so that the search would yield a broader set of organizations. The search in late 2023 yielded 5279 documents published between 1993 and 2023. This included all types of documents indexed by Web of Science, including articles, proceedings, and book chapters. While a majority of Web of Science publications are peer-reviewed, some types of publications, such as letters and conference abstracts, may not have been peer-reviewed. Documents were divided into pre-pandemic (1993–2019) and post-pandemic (2020–2023) periods. The pandemic was used as a chronological marker to examine whether there were shifts in research focus and publication data, regardless of when the underlying data was collected. Author 2 analyzed literature from the first period, Author 1 from the second. Human subjects’ approval was not needed for bibliometric analysis.

#### Data cleanup and preparation

We used VOSviewer, an open-source software that analyzes bibliometric networks and visualizes how documents are related. VOSviewer performs co-authorship, country, organization, and keyword co-occurrence analysis. After filtering terms by year, full records from search results were retrieved as text files for VOSviewer analysis. A thesaurus file was generated for data cleanup after importing files into VOSviewer.

Keywords included identical terms with variant spellings (e.g. healthcare, healthcare, health care). To avoid duplication, the team created a thesaurus file for each period where words with same meanings were merged. Author 1, Author 2, and Author 5 determined words to merge by highlighting them in red and choosing one word to represent them. The new keyword remained consistent for both periods, ensuring comparative analyses before and after COVID were not compromised. Words irrelevant to the study’s scope, such as methodological terms, were marked for removal by adding “remove” next to the keywords. After keywords were labeled correctly by all team members, they were filtered by selecting keywords labeled “removed” and non-highlighted keywords. Keywords not included in the thesaurus file were removed. This refined keyword list was then imported into VOSviewer for co-occurrence analysis.

### Publication trends, country and organization analysis

To identify and analyze the number of publications, countries, and organizations, we exported VOSviewer data into a spreadsheet, computed counts, and ranked the top 10 countries and organizations with the highest number of publications.

To identify collaborations within and between countries, we performed co-authorship analysis. For country analysis, “co-authorship” was selected as the type, and the unit was set to “countries” in VOSviewer’s co-occurrence analysis dialog. The full counting method was used, with the other parameters at default. A minimum of five documents per country were specified. Bibliometric visualizations were generated.

### Keyword and co-occurrence analysis

To identify the keywords occurring together in publications, we performed a co-occurrence analysis. Co-occurrence analysis indicates the relationship between keywords based on the number of times they appear together in shared documents.^41,42^ Each keyword required a minimum of three occurrences for inclusion in the co-occurrence analysis. We employed a full counting method that equalized the weighting of all keyword co-occurrences. All other default parameters in VOSviewer were retained. Co-occurrence visualizations and keyword clusters were generated.

### Comparative analysis

To identify similarities between themes in 1993–2019 and 2020–2023, we manually compared data in a spreadsheet. Two columns were created: one with keywords labeled “Pre-COVID” (1993–2019), and another with “COVID” (2020–2023). Duplicated keywords were highlighted using conditional formatting to identify topic similarities and differences between periods. After identifying similarities and differences, keywords were categorized by major themes. The number of occurrences of each keyword was analyzed. To compare countries and organizations conducting healthcare burnout research before and after 2019, a similar procedure was employed.

## Results

### Burnout in healthcare is a longstanding problem researchers continue to investigate

**Figure 2.**
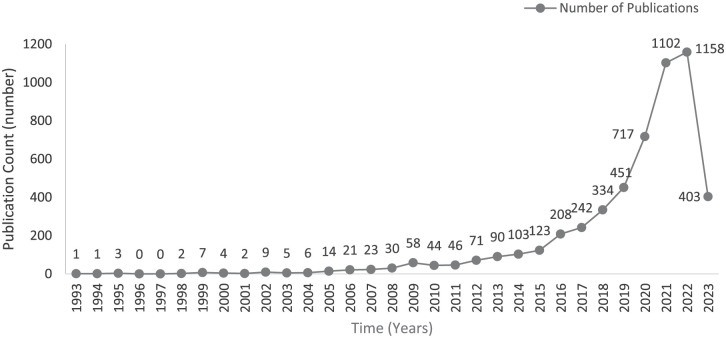
All publications indexed by Web of Science on burnout in the healthcare sector between 1993 and 2023. Source: Author’s own work.

To identify whether burnout in healthcare is a longstanding problem, we analyzed trends in healthcare burnout topics that have been investigated over time by evaluating the growth or decline in publications between 1993 and 2023. We retrieved 5279 scholarly publications addressing burnout among HCWs from the Web of Science. We found minimal studies on this topic during 1993–2008, with fewer than 30 documents (Figure 2). In 2009, the number of publications increased to 58 but dropped to 46 by 2011. Subsequently, the number of publications rose steadily, peaking at 1158 in 2022. Of all documents published across all periods, 36% (*n* = 1899) were published between 1993 and 2019. However, most publications were in the post-COVID-19 period between 2020 and 2023 (*n* = 3380). These trends indicate that burnout topics in healthcare have been investigated for a long time, with a gradual increase in interest in the topic, and COVID-19 creating a surge in interest in investigating burnout.

### Burnout in healthcare is a widespread problem investigated by researchers across organizations worldwide

**Figure 3.**
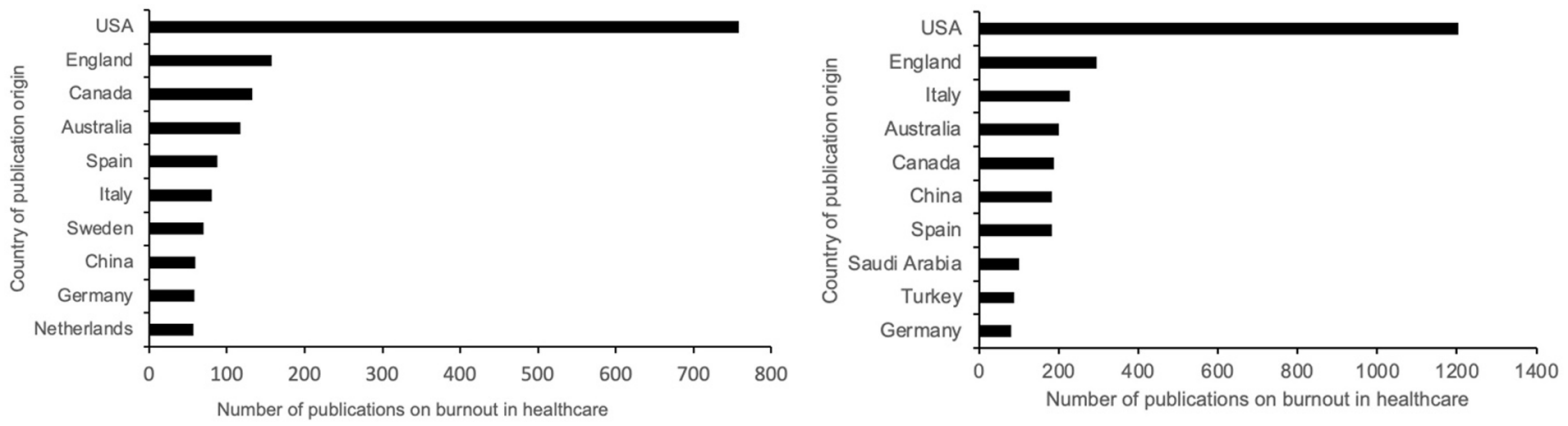
(a) Top 10 countries with the highest number of publications on burnout in healthcare between 1993 and 2019. (b) Top 10 countries with the highest number of publications on burnout in healthcare between 2020 and 2023. Source: Author’s own work.

To identify whether burnout in healthcare is a widespread problem, we analyzed the number of countries and organizations investigating burnout between 1993 and 2023, and the growth or decline in these trends. We found that 1899 documents published before COVID-19 were distributed across 48 countries. Between 1993 and 2019, publications from North and South America originated from two countries each. 23 European countries, 13 from Asia, two from Africa, and two from Oceania, made up the rest. Post-pandemic, countries researching healthcare burnout increased to 83. Three were from North America, seven from South America, 29 from Europe, 29 from Asia, eight from Africa, and two from Oceania. Figure 3(a) displays the top 10 countries with the highest number of publications from 1993 to 2019, with United States leading with approximately 40% of the publications (*n* = 758), followed by Great Britain (*n* = 158) and Canada (*n* = 133). Figure 3(b) displays top 10 countries with the highest number of publications between 2020 and 2023 with United States at 36.7% (*n* = 1205), followed by Great Britain (*n* = 296), and Italy (*n* = 228).

Our findings show that 179 organizations conducted research on burnout from 1993 to 2019, with 44 participating in at least five publications, 72 in six to nine, and 62 in at least 10. The number of organizations increased to 416 in 3 years after the pandemic, with 96 participating in at least five publications, 180 contributing to between six and nine, and 139 participating in at least 20 publications.

Our findings demonstrate that healthcare burnout research occurs worldwide across multiple organizations, suggesting that the problem of burnout and the efforts to investigate it are widespread and global.

### Early research efforts (1993–2019) toward addressing burnout were isolated to collaborations within countries, but recent post-COVID-19 efforts (2020–2023) have seen increases in cross country and cross continental collaborations

**Figure 4.**
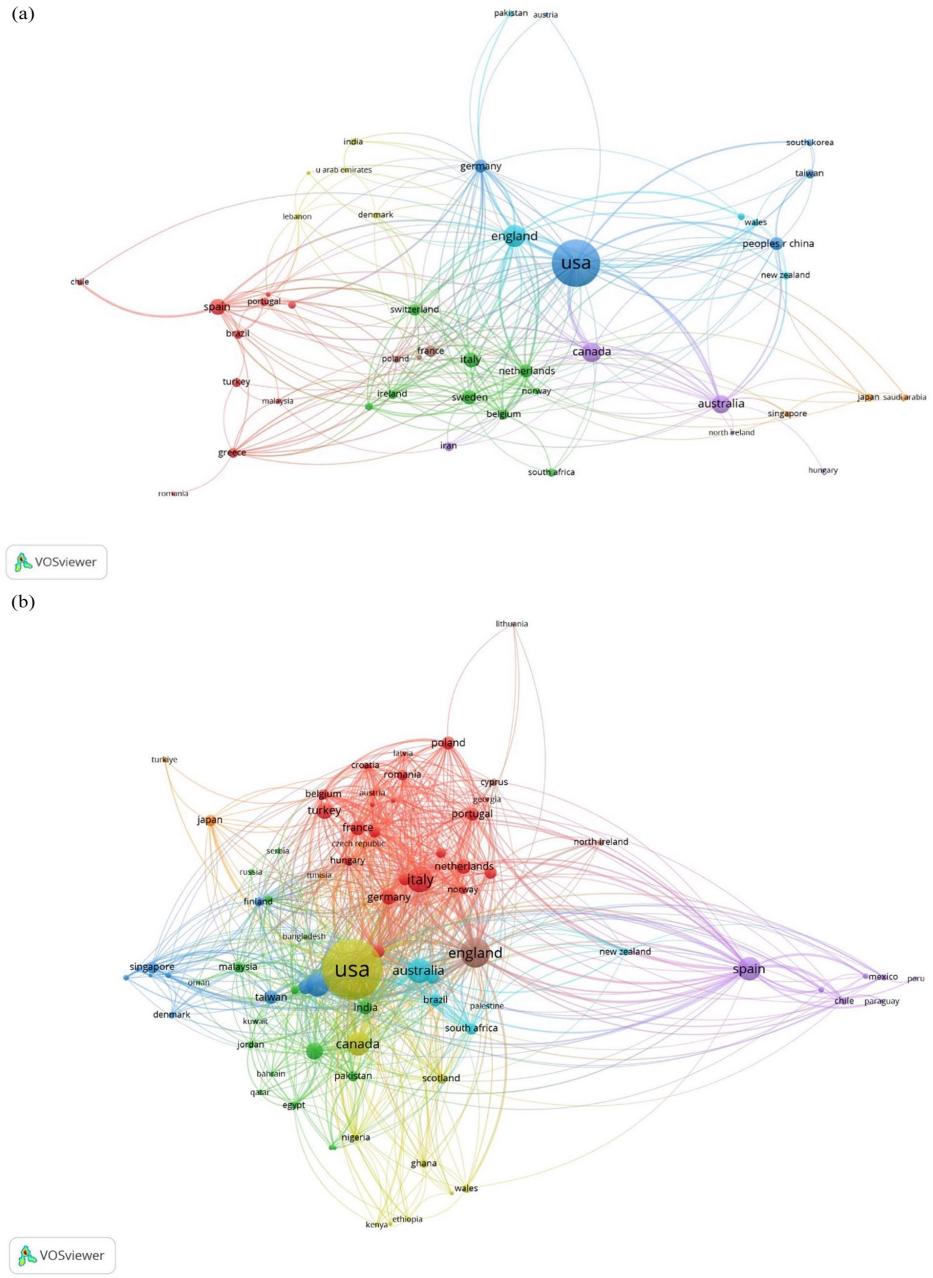
(a) Co-authorship bibliometric network representing countries publishing on burnout in healthcare between 1993 and 2019. (b) Co-authorship bibliometric network representing countries publishing on burnout in healthcare between 2020 and 2023. Source: Author’s own work.

To identify the extent of worldwide collaborative efforts in addressing burnout, we analyzed the co-authorship networks within and between these countries. Figure 4(a) illustrates the co-authorship network among countries in 1993–2019, showing co-authorship links and clusters occurred more within a country than between countries. This trend changed in studies between 2020 and 2023 which show more links and collaborations between countries (Figure 4(b)). Our findings demonstrate the growth in international collaborations in investigating and developing global solutions to burnout.

### System and organizational factors are persistently studied in the context of healthcare burnout

To determine whether there are persistent problems contributing to burnout that have been examined by researchers over an extended period, we analyzed keyword similarities between the periods 1993–2019 and 2020–2023 and categorized them into themes. The findings revealed that 492 keywords were similar between the two periods. The themes based on the categorization of similar keywords across time periods included the following:

- Healthcare professionals: This category encompasses various types of HCWs who dealt with burnout-related problems. Keywords include advanced practice nurses, anesthesiologists, clinicians, emergency medicine physicians, medical residents, medical students, oncologists, physician assistants, psychiatrists, registered nurses, and surgeons.
- Leadership and management: Keywords in this category relate to management from supervisors, leadership opportunities, and organizational problems. Examples include coaching, leadership, management, managers, organizational change, organizational commitment, organizational culture, organizational support, supervisor support, transformational leadership, workplace bullying, and workplace empowerment.
- Patient safety: Keywords in this category relate to the quality of care and patient safety, including care, care professionals, clinical practice, patient care, patient outcomes, patient safety, patient satisfaction, patient-centered care, quality improvement, quality of care, safety climate, and service quality.
- Working conditions: Working environment characteristics and related problems were addressed, including climate, environment, ergonomics, flexibility, gender differences, organizational support, staffing, work conditions, workload, workplace safety, workplace stress, and workplace violence.
- Specific populations: The literature includes information about populations dealing with burnout, such as families, family caregivers, and junior doctors. It also covers characteristics of patients, HCWs, including children, older people, parents, and veterans.
- Coping strategies: Researchers have included strategies to reduce the effects of burnout, such as cognitive therapy, compassion fatigue, compassion satisfaction, emotional regulation, mindfulness, self-care, self-compassion, stress management, and yoga.

These findings suggest that system and organizational factors leading to burnout are persistent, and researchers have been studying these factors in specific populations, its impact on safety, and interventions such as coping strategies to address burnout due to these factors.

### COVID-19 has introduced new research themes in healthcare burnout such as workplace adaptations, workplace aggressions and emerging technology

**Figure 5.**
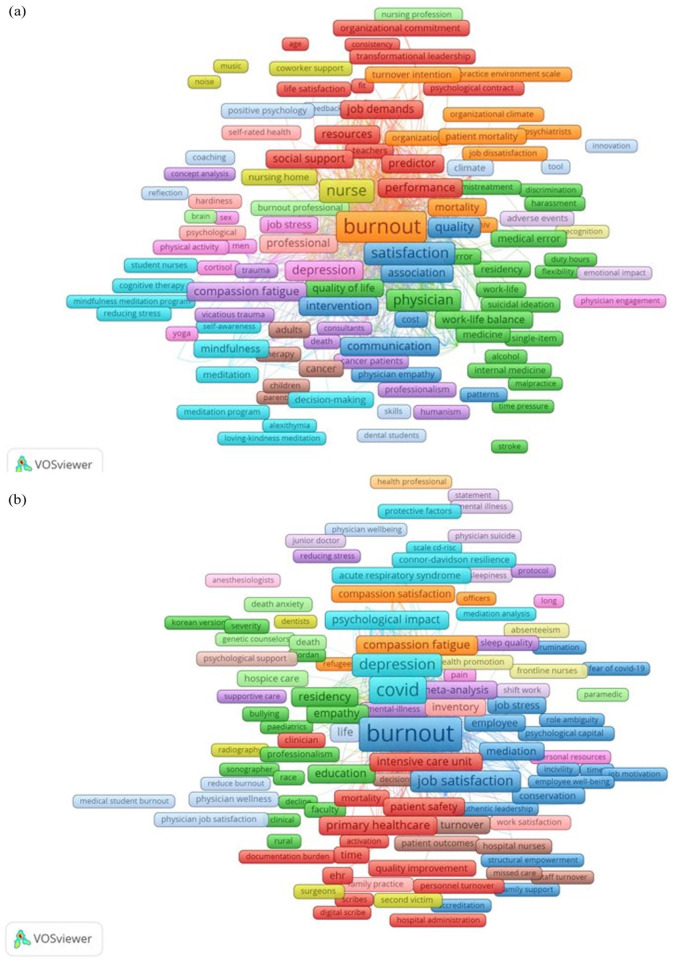
(a) Co-occurrence bibliometric network representing keywords featured in the literature on burnout in healthcare between 1993 and 2019. (b) Co-occurrence bibliometric network representing keywords featured in the literature on burnout in healthcare between 2020 and 2023. Source: Author’s own work.

To identify whether COVID-19 introduced new research themes in healthcare burnout, we analyzed keyword co-occurrences to identify top keywords pre- and post-COVID, compare them, and identify newer keywords that emerged between 2020 and 2023. We found that burnout, stress, and nurse were the top keywords by frequency (Table 1 and Figure 5(a)) between 1993 and 2019, whereas COVID-19, burnout and stress emerged among the top keywords (Table 2 and Figure 5(b)) between 2020 and 2023. “Healthcare professional” was among the top keywords in both periods, appearing more frequently than specific terms such as “nurse.” Co-occurrence analysis uncovered differences in burnout-related themes across periods, in particular showing new research themes during 2020–2023:

- Aggressions: Abusive supervision, sexual harassment.
- COVID: Adaptation, COVID-19, epidemic, fear, lockdown, pandemic, and epidemic.
- Technology: Artificial intelligence, digital health, machine learning, social media, telemedicine, virtual reality.
- Types of HCWs: Frontline workers, neonatal intensive care units, radiographers, paramedics, and neurosurgery.

**Table 1.** Top 10 keywords featured in the literature on burnout in healthcare between 1993 and 2019.

No	Keyword	Number of occurrences	Total link strength
1	Burnout	1282	9439
2	Stress	471	3791
3	Nurse	404	3355
4	Satisfaction	334	2664
5	Job satisfaction	294	2526
6	Physician	243	1844
7	Care	239	1779
8	Health	205	1655
9	Healthcare professional	190	1578
10	Impact	173	1447

**Table 2.** Top 10 keywords featured in the literature on burnout in healthcare from 2020 to 2023.

No	Keyword	Number of occurrences	Total link strength
1	Burnout	2285	16,741
2	Covid	920	6481
3	Stress	750	6213
4	Healthcare professional	704	5508
5	Nurse	633	5330
6	Mental health	559	4735
7	Impact	447	3848
8	Depression	421	3689
9	Physician	359	2887
10	Care	353	2654

These findings indicate that COVID-19 introduced new research themes linked to healthcare burnout such as COVID-19 related workplace adaptations, workplace aggressions, and emerging technology such as virtual reality.

## Discussion

Our findings demonstrate that the problem of burnout in healthcare is a longstanding and widespread problem investigated worldwide, with increased global collaborations after COVID-19. Although early research efforts (1993–2019) toward addressing burnout were isolated to collaborations within countries, recent post-COVID-19 efforts (2020–2023) have seen increases in cross-country and cross-continental collaborations. System and organizational factors such as poor workplace conditions, leadership and management appear to be persistent problems studied both pre- and post-COVID-19. COVID-19, however, has compounded the problem and has now catalyzed new burnout research themes, including workplace adaptations such as remote work due to COVID-19, its impact on burnout, workplace violence, and aggression and its impact on healthcare worker safety, and emerging technologies such as virtual reality that may help reduce burnout.

The gradual increase in publications since 1990s along with the surge during COVID-19, implies that burnout has been a longstanding problem, with studies investigating its extent, its impact on individuals and organizations, and interventions to prevent it. Publication trends demonstrate a steady increase in burnout-related publications between 2011 and 2022, and then a surge from 1899 publications between 1993 and 2019 to 3380 between 2020 and 2023. The gradual increase in publications between 1993 and 2019 aligns with the shift from foundational to evaluative burnout studies, with development of major measures and its psychometric validation^3,4^ such as MBI, development of theoretical frameworks explaining development of burnout,^43–46^ and extension to different occupations within the healthcare sector^47^ and cultural contexts.^48^ By late 2019, scholars were still debating limitations in burnout evaluation measures. The surge in studies between 2020-2023 stemmed from the pandemic’s adverse impact on burnout prevalence^49^ and studies assessing the well-being of frontline clinicians caring for COVID-19 patients.^28,30,50–53^ We believe that COVID-19 unraveled accumulated workplace stress when HCWs faced significant resource constraints, shifting workplace conditions, and risks to themselves and their families. Scholars responded with increased research on HCWs’ well-being and burnout mitigation. Furthermore, the National Academies of Medicine’s 2019 report^1^ highlighted workplace burnout challenges and called for research studies and interventions to combat clinician burnout. The onset of the pandemic in March 2020 led to a significant number of studies on burnout in healthcare between 2020 and 2023.

The study of healthcare burnout in 83 countries and 416 organizations over time suggests that burnout is a widespread problem. The significant increase in the number of countries (48–83 countries) and organizations (179–416) studying burnout in healthcare reveals its emergence as a global phenomenon. The rise in organizations studying burnout signals awareness of its impact and the priority and commitment to developing interventions. This widespread nature of burnout, while problematic, offers promising opportunities to comprehensively study, and systematically devise interventions based on data collection worldwide across a multitude of organizations. It also raises questions about the impact of culture, organizational design, healthcare worker training, and geographical characteristics on well-being and occupational burnout.

While pre-pandemic healthcare burnout research was limited to 48 countries, with within-country collaboration more prominent, the post-pandemic research spans 83 countries with increased cross-country and cross-continental collaboration. This increased international collaboration shows potential for developing global solutions. This international attention to burnout is long overdue and emphasizes burnout as a healthcare management challenge requiring global, sustainable, universal interventions for prevention.

The persistent occurrence of 492 keywords and its corresponding themes across both periods suggest that fundamental organizational and management factors remain challenges. Amongst other themes, two themes “Working Conditions” and “Leadership and Management” occurring in both periods reflect long-standing system-level and organizational management challenges contributing to burnout.

Workplace conditions and job satisfaction were among the top keywords in healthcare burnout literature between 1993 and 2019. The “working conditions” theme includes keywords representing physical working conditions, workplace violence, workload, technology adoption, workplace culture and climate, work-life balance, and job satisfaction. Between 1993 and 2019, for example, studies examined the relationship between burnout and job satisfaction among HCWs using MBI and other tools.^54^ Research showed that increased absenteeism, decreased job satisfaction, staff turnover were linked to burnout with potential to impact personal life,^23^ but scholars also called for more studies on how specific aspects of job satisfaction contributed to burnout.^27^ Studies between 2020 and 2023 focused on workplace conditions and behavior, adaptation to COVID and technology, and healthcare professionals. Psychosocial work environment factors including job demands, resource constraints^55^ organizational and job content and interpersonal relations and leadership are shown to influence burnout.^56^ During COVID-19, increased workload due to rising patient volume, personal protective equipment (PPE) shortages, and pandemic uncertainty exacerbated burnout in healthcare.^57–60^ In particular, new clinicians experienced significant workload due to challenges transitioning to their clinical roles, largely due to online clinical training during the pandemic and limited real-world practice.^61^ Additionally, personnel shortages^62–65^ forced HCWs to learn and adapt new skills outside their core responsibilities, increasing workload and role ambiguity, leading to burnout. These shortages impacted not just physicians and nurses but allied healthcare professionals and all types of healthcare workers, including those who performed direct patient care and those who did not.^65–70^ Additionally, healthcare workers were directly impacted by COVID-19 infections, either to themselves or their family, further exacerbating shortages and burnout.^71^ The healthcare workforce shortage is only expected to become more acute, with WHO predicting a global deficit of 18 million healthcare workers by 2030.^15^

Research has also explored working conditions in relation to workplace violence and work-life balance. US Bureau of Labor Statistics (BLS) data shows evidence of increasing workplace violence among HCWs. Approximately 20,050 workers experienced non-fatal workplace violence in 2020, with 76% in the healthcare and social assistance industry. US BLS data indicate that healthcare practitioners and technical occupations accounted for 23% of non-fatal workplace intentional injuries (8590 injuries) caused by another person and requiring at least 1 day away from work in 2020.^72^ In the healthcare and social assistance industry, approximately 15,210 cases of non-fatal workplace intentional injuries by another person were reported,^73^ rising to 28,970 between 2021 and 2022. Scholars report that workplace violence among health professionals is linked to negative outcomes affecting their quality of life and well-being.^74–77^ In addition, there is a demonstrated relationship between work experiences,^78^ burnout, and psychological safety.^79^ Psychological safety is the feeling of “reduced interpersonal risk”^78^ in work environments. Psychologically safe work environments tend to not only engage workers more,^80^ but also help with managing stress and providing avenues for mitigating workplace violence.^81,82^ These findings suggest the need for both short- and long-term measures to address undesirable workplace conditions.

Studies have also examined lack of work-life balance as an undesirable workplace condition and its implications for burnout. For example, studies have shown that female physicians may experience increased burnout when they have children. In addition to working as health professionals, they perform uncompensated domestic work.^83^ McMurray et al. found that spousal support in household tasks positively influences burnout prevention.^84^

The “leadership and management” theme is also persistent across time. Our findings demonstrate that both before and after COVID-19, concerns in well-being and burnout due to leadership and organizational management surface in the literature.^85–88^ Nagle et al. indicate that organizational factors, such as inadequate support and stressful work environments, can contribute to burnout.^27^ Studies also investigated organizational support as a critical moderating factor for work performance, with positive support reducing effects of work stressors on burnout.^89^ Our findings also correspond with Shanafelt and Noseworthy’s research, showing that burnout determinants associated with workload, efficiency, flexibility, culture, values, community, control, purpose, and organizational support consistently affect healthcare worker well-being.^90^ This suggests a need to revisit design and management of organizational support and work systems and develop interventions. Furthermore, the sixth theme “coping strategies” has been investigated since the 1990s, but keywords related to burnout interventions seem to focus more on individual interventions.^53^ This raises questions on the contribution of organizational factors to burnout, development of organizational interventions, and assessment of its impact and efficacy to prevent burnout.^1^

These workplace contributors point to persistent and systematic challenges associated with HCWs’ burnout and well-being. Factors affecting workplace conditions, well-being, and burnout are multidimensional, and may reflect larger system-level challenges requiring further investigation to understand why it is persistent, and what interventions can help address these challenges. We believe that these workplace stressors accumulate over time, become persistent, and require systematic and comprehensive organizational mitigation strategies and interventions to address underlying undesirable workplace conditions. Our findings on workplace conditions and increased work demands and their association with burnout are particularly important and timely, given the healthcare workforce is struggling with workforce retention and preservation.^15,67^ Future work should investigate the relationships between working conditions and burnout, mental health, and well-being, as well as their role during large-scale crises, to develop or tailor organizational interventions that identify, prevent, and mitigate the impact of burnout among HCWs.

In investigating whether COVID-19 has introduced new research themes in healthcare burnout, we observe that (1) COVID-19 is a key topic between 2020 and 2023 which is not surprising; studies investigated how COVID-19 workplace conditions impacted burnout and stress between 2020 and 2023; (2) workplace aggressions, a specific category within workplace violence occurs as a keyword between 2020 and 2023, and (3) technology keywords such as artificial intelligence (AI), virtual reality (VR) and social media emerge between 2020 and 2023.

COVID-19 significantly altered the healthcare workplace and demanded workplace adaptations. This triggered high levels of burnout among HCWs and increased the number of research studies examining the link between COVID-19 and burnout in healthcare. Ibrahim et al. show that poor professional quality of work life factors such as high workload, low resources and psychological stressors significantly impacts burnout.^91^ US Centers for Disease Control’s (CDC) Quality of Worklife Survey indicated an increase in burnout from 32% in 2018 to about 46% in 2022, and by 2021,^92^ 40% of HCWs intended to leave within 5 years.^93^ A nationwide study in the US of 20,947 HCWs also found that 43% reported work overload and 49% experienced burnout.^94^ This points to the impact on well-being from managing a large scale crisis, accounting for the increase in research studies to assess prevalence and develop interventions for burnout.

Although workplace violence has been persistent in healthcare^95–98^ and was used as a keyword prior to 2020, workplace aggression emerges as a theme after COVID-19,^74,99,100^ with an increase in keywords such as “bullying,” “abusive supervision,” and “sexual harassment,” perhaps due to anxiety, uncertainty, and isolation. US CDC’s Quality of Worklife Survey indicated a doubling of workplace harassment rates from 6% to 13% in 2022.^92^ Workplace aggression also extended beyond organizational factors to interactions with patients. Scholars report that HCWs experiencing constant pressure, high levels of stress, and bullying could develop low self-esteem, problems perceiving accomplishments, emotional exhaustion, and low job satisfaction, leading to burnout.^31^ We think the theme of workplace aggression among HCWs reflects a larger societal trend toward aggressive acts in public spaces. While some aggressions are visible, knowledge on invisible and unreported aggressions remains limited.^101–103^ Invisible and unreported aggressions are potentially more consequential for well-being as they can accrue, especially if HCWs ignore or hide them but continue to experience stress or trauma. These findings highlight workplace aggression as a critical, underexplored factor and emphasize the need to investigate and address invisible and unreported workplace aggressions.

Another emerging research theme after COVID-19 is the technology cluster with keywords including social media, telemedicine, VR, and AI. Social media’s impact on HCWs’ well-being is mixed. It raised mental health awareness but was also misused for cyberbullying of HCWs by patients.^104^ La Regina et al. analyzed healthcare social media posts and reported that 74% of posts and comments were negative, containing criticisms, offensive language, and threats.^104^ Researchers urge attention to this problem, given the severe psychological consequences, from anxiety to depression and suicide, among health professionals.

In addition to social media, emerging technologies such as telemedicine, VR, machine learning, and AI occurred as keywords. We believe telemedicine emerged as a keyword due to its significant use during the pandemic,^105,106^ while VR and AI as themes may reflect studies assessing these technologies for early prediction and interventions to improve clinicians’ well-being on a path to burnout.^107–111^ For example, Beverly et al. used VR to simulate an environment where frontline health professionals during COVID-19 could relax during breaks.^112^ Participants perceived stimuli from nature, such as looking at the sun, listening to birds, and the sounds of leaves. Results showed a significant reduction in stress post-simulation. These technological interventions point to the potential for both group-based and individual approaches to improve well-being. Our findings suggest that emerging technologies such as VR and AI can be used for burnout prevention and management. In particular, we believe that organizational interventions integrating these technologies could be effective solutions for preventing burnout. Similar to healthcare organizations, including meditation rooms, they can purposefully design well-being spaces by integrating these technologies into their physical infrastructure for holistic interventions.

### Limitations

The time periods in our study were not equal: 26 years between 1993 and 2019 and only 3 years between 2020 and 2023. Given our objective to understand persistent factors contributing to burnout and new research themes after COVID-19, this selection was necessary. Additionally, 2024 data was not captured in this analysis. Publication dates of the studies included in this analysis may not reflect the timing of data collection, and some studies published after March 2020 may have been based on pre-pandemic data. We used only Web of Science database and did not use any gray literature for our study. Given Web of Science offers comprehensive citation data, we think we captured a significant portion of the healthcare and burnout literature, but the exclusion of gray literature and the use of only one database may have impacted the comprehensiveness. We provide a definition of burnout based on the MBI,^4^ but we acknowledge that there are alternative definitions, characterizations, and instruments to assess burnout that can provide a complementary view. A final limitation is that a bibliometric analysis provides the scientific landscape in a topical area and potential research directions but does not provide an in-depth review of each study.

### Future work and design implications

We present implications and recommendations for organizations, technology design, and future research (Table 3). Burnout has significant consequences for HCWs, their families, patients, healthcare organizations, and society, demanding urgent research and development of interventions to prevent, reduce, and mitigate burnout in healthcare.

**Table 3.** Implications and recommendations for organizational design, technology design, and future research on burnout in healthcare.

Theme	Recommendations
*Organizational implications and recommendations*	
Workplace aggressions	• Establish reporting systems • Develop escalation pathways for timely reporting and mitigation of aggravating workplace factors • Establish clear policies and action steps
Undesirable workplace conditions	• Assess and improve staffing and workload through evidence-based retention strategies • Leadership training on well-being of employees and corresponding management and leadership strategies • Well-being metrics along with safety metrics for reporting and auditing • Organizational culture change and dedicated resources for well-being • Policies, processes and structures to enhance psychological safety • Crisis preparedness protocols for rapid and effective adaptations
Prevention and mitigation of burnout	• Integrate burnout screening protocols • Decompress and recovery spaces • Occupational recovery and rehabilitation programs • Scheduled decompress periods in a shift
*Technological implications and recommendations*	
Identification and prevention of burnout	• Develop early warning systems for proactive identification and prevention of burnout • AI based support systems for activity monitoring and scheduled activity breaks • VR based wellness programs
Mitigation and recovery from burnout	• Digital wellness platforms provided by the organization • Technology enabled recovery spaces • Telehealth support to facilitate recovery • Automated tools to assist with task demands and workload
*Future research recommendations*	
Invisible workplace aggressions	• Understand determinants of workplace aggressions and challenges in identification, assessment and reporting • Understand
Technology interventions	• Assess the effectiveness of technology based wellness programs in reducing or preventing burnout • Understand how integration of virtual care systems influenced burnout • Identify how technology and automation can assist in reducing workload and shift burden from employees • Develop and understand the utility of technologies and platforms to support crisis specific situations
Organizational factors	• Understand the effectiveness of current organizational interventions • Assess the impact of organizational characteristics, leadership skills and attributes on employee well-being and burnout • Understand effects of workload and staffing shortage • Assess the impact of psychological safety for different types of healthcare workers and different types of healthcare organizations to understand its contributing or mediating role in burnout • Identify impact of work system redesign including the effectiveness of different types of work models such as hybrid work models in healthcare in improving workforce retention • Investigate new models of shift rotation and staff workload especially during crisis situations
Global evidence	• Assess the globally available evidence on determinants and prevention of burnout • Identify global best practices for burnout prevention • Understand influence of culture/work culture on burnout
Health worker population	• Assess burnout and well-being among other types of healthcare workers who perform direct and indirect patient care • Assess burnout and well-being among allied health professionals who interface with healthcare systems, but do not directly work in one • Compare and understand the contributors and mediators of burnout across diverse healthcare worker populations

Organizational interventions must be effective and sustainable to promote early identification of burnout pathways and to eliminate its sources or mitigate its effects. Organizations should also allocate resources and setup occupational recovery programs to help with recovery and rehabilitation from burnout. Furthermore, interventions should span across holistic and permanent solutions, such as physical spaces to improve well-being or redesign of work policies, as well as periodic evidence-based activities, such as group recreation or training. Most importantly, organizations must ensure healthcare workers feel “reduced interpersonal risk” or psychologically safe in their work environments^78^ for any intervention to be effective. Technology interventions for assessing and mitigating burnout is emerging and shows promise for creating individualized plans to improve well-being and reduce burnout. Future research should aim to assess and understand contributors to burnout among all types of healthcare professionals, such as pharmacists, dietitians, social workers, respiratory therapists, and others, and develop interventions tailored to their needs. Future research in healthcare burnout should focus on investigating and developing effective interventions to address burnout by leveraging international collaboration and advanced technology such as Artificial Intelligence.

Burnout is an occupational phenomenon requiring strong management and organizational interventions to reduce it among HCWs. Our insights into urgent, persistent, and system-level factors impacting burnout can help develop targeted organizational and technological interventions and inform policy decisions about healthcare workforce preservation. Our findings on challenges and research directions triggered by COVID-19 can help develop crisis-specific interventions for burnout and well-being and prepare healthcare systems to become resilient to future crises. Revisiting work system design and working conditions in the healthcare system will help root out system-level challenges that lead to burnout and prevent reactive, temporary solutions.

## Conclusions

Burnout among HCWs requires urgent attention to ensure provider well-being, maintain patient safety and prevent organizational challenges. Our findings indicate that burnout is a long-standing and widespread problem, with workplace conditions and organizational factors such as leadership and management, remaining persistent challenges. Furthermore, workplace violence and workplace aggression are increasingly associated with burnout. Emerging technologies such as artificial intelligence and virtual reality hold promise as interventions for preventing and mitigating burnout. Our findings indicate a need to understand how work system design could help identify, prevent, or mitigate burnout and which organizational interventions might be sustainable and effective in addressing burnout. Future studies should also explore how emerging technologies can deliver effective interventions at the individual, group, and organizational levels for a more holistic solution to HCWs’ burnout.
